# Pantoea agglomerans in Equine Ulcerative Keratitis: Prevalence and Comparative Efficacy of Four Topical Antiseptics

**DOI:** 10.1111/vop.70044

**Published:** 2025-06-25

**Authors:** Frederik Heun, Jessica Meißner, Ann‐Kathrin Schieder, Bernhard Ohnesorge, Claudia Busse

**Affiliations:** ^1^ Clinic for Horses University of Veterinary Medicine Hannover Hannover Germany; ^2^ Department of Pharmacology, Toxicology and Pharmacy University of Veterinary Medicine Hannover Hannover Germany; ^3^ LABOKLIN GmbH & co. KG Bad Kissingen Germany; ^4^ Clinic for Small Animals, Department of Ophthalmology University of Veterinary Medicine Hannover Hannover Germany

**Keywords:** eye, horse, hypochlorous acid, N‐acetylcysteine, polyhexanide, PVP‐iodine

## Abstract

**Objective:**

To determine the minimal bactericidal concentration (MBC) and effective contact time of four topical antiseptics—polyhexanide, povidone‐iodine (PVP‐I), hypochlorous acid (HOCl), and N‐acetylcysteine (NAC)—against *Pantoea agglomerans* , a pathogen frequently isolated in equine ulcerative keratitis.

**Animals Studied:**

Over a 17‐month sampling period, clinical isolates were collected from horses with ulcerative keratitis. The most frequently isolated strain (*Pantoea agglomerans, n* = 14) was selected for in vitro analysis.

**Procedure(s):**

All isolates were used to determine the MBCs of the four antiseptics. Each was tested in triplicate at serial dilutions per isolate. Additionally, the requisite contact time for a bactericidal effect was evaluated at a supratherapeutic dilution for each substance with each isolate at defined time points ranging from 15 s to 5 min.

**Results:**

The MBCs of polyhexanide, PVP‐I, HOCL, and NAC were 3.2 ppm (0.00032%), 16 ppm (0.0016%), 0.8 ppm (0.00008%), and 3200 ppm (0.32%), respectively. Polyhexanide (6.4 ppm), PVP‐I (64 ppm), and HOCL (6.4 ppm) were effective within 15 s. NAC (6400 ppm) required 1–2 min to achieve bactericidal effects.

**Conclusions:**

All antiseptics tested demonstrated efficacy against *P. agglomerans* . Polyhexanide, PVP‐I, and HOCl achieved rapid bactericidal activity, while NAC required higher concentrations and longer exposure. These results support the use of these agents—particularly the faster‐acting three—as potential alternatives to antibiotics in treating equine ulcerative keratitis. They may aid the reduction of antibiotic use in line with the One Health approach.

## 1 Introduction

Ulcerative keratitis in horses is frequently associated with local bacterial infection [1, 2]. Studies of equine ulcerative keratitis in recent decades, for example, in Belgium [1], the United States (US) [3, 4], and Japan [5], have shown that *Staphylococcus* spp., *Streptococcus* spp., and *Pseudomonas* spp. are the primary genera involved. Most publications suggest that the microflora of healthy equine eyes is dominated by Gram‐positive bacteria [6, 7, 8]. However, a recent study from the United States using culture‐independent, DNA‐based detection methods showed that Gram‐negative bacteria are more common on the ocular surface [9]. *Pantoea agglomerans* is a Gram‐negative bacterium from the family *Erwiniaceae*. It is infrequently isolated from both healthy equine eyes and those with bacterial corneal ulceration [1, 2, 3, 6, 10]. A recent study from Germany, which looked at ocular surface swabs from equine eyes with various diseases, has shown that *Pantoea agglomerans* is the most common Gram‐negative pathogen from diseased equine eyes [11, 12]. Interestingly, *Pantoea agglomerans* was also the most common Gram‐negative pathogen in the aforementioned study on ulcerative keratitis from Belgium [1], while it was only very rarely detected in the other studies mentioned above. Its facultative pathogenicity and ability to infect corneal ulcers have been described in human medicine [13, 14]. Nevertheless, the frequent occurrence in healthy eyes of horses [10] and donkeys [15] suggests that initial damage to the corneal defense mechanism is necessary for infection to occur. To date, no studies on equids have specifically addressed ulcerative bacterial keratitis caused by *Pantoea agglomerans* in horses.

Antibiotic therapy remains the primary approach for managing corneal bacterial infection [2]. However, the increasing resistance of pathogens to commonly used first‐line antibiotics [1] necessitates the exploration of alternative bactericidal treatment regimes.

Povidone‐iodine (PVP‐I) is a widely used disinfectant [16] and well‐tolerated on the ocular surface at concentrations ranging from 0.05% to 0.5% [17]. The effective contact time is concentration‐dependent and varies significantly with the type of bacteria being treated, leading to heterogeneous recommendations for contact time in human medicine, which range from 30 s [18] to 3 min [19, 20]. The bactericidal effect of PVP‐I is primarily due to the release of free iodine, which generates free radicals that destruct membrane proteins [16]. This effect can also impact the corneal epithelium in a concentration‐dependent manner, for example, 5% PVP‐I damages the epithelium of rabbit eyes after local application. This has led to restrictions in its use for prolonged local therapy in higher concentrations [21].

Hypochlorus acid (HOCL) solutions are potent disinfectants routinely used in the treatment of infections of the eye and periocular tissues in humans, such as blepharitis [22]. Originally, HOCL is part of the innate immune system, where it is produced, for example, by neutrophils; it kills bacteria by oxidizing various biological molecules, causing bacterial destruction [23]. HOCL has been shown to reduce bacterial load on the periocular surface within 20 min by > 99% in humans [24]. However, the direct comparison of HOCL with PVP‐I on ocular surface yielded variable results, that is, in some cases the results were comparable [25], in others HOCL was less effective in reducing colony‐forming units (CFU) in the human eye [26]. Nevertheless, HOCL consistently resulted in less discomfort for the patients following local lavage [25, 26, 27]. In veterinary ophthalmology, HOCL‐containing sprays have been reported to positively impact healing of bacterial corneal ulcers in bovines [28].

Polyhexamethylene biguanide (PHMB) is a commonly used antiseptic agent prior to ocular surgery [29, 30]. It has been shown to be as effective as PVP‐I, causing fewer side effects, such as irritation [31]. PHMB, a cationic antiseptic, interacts with the phospholipids of cell membranes as well as with lipopolysaccharides or peptidoglycans, resulting in dysfunction and destruction of the cell membrane [32]. Although PHMB exhibits a potent cytotoxic effect in vitro, this effect has not been demonstrated in intact corneal epithelium, suggesting that PHMB is safe for use in ocular disinfection [33].

N‐acetylcysteine (NAC) had a primary antibacterial effect, demonstrated against endodontic pathogens from humans [34], various bacteria from the dog's external ear canal [35], and recently against the most important pathogens in ulcerative keratitis in dogs and cats [36]. A possible mechanism of action includes the direct reducing ability of the molecule [37], or its influences on the production of extracellular polysacarides [38], which could impact bacterial cells. NAC has shown the ability to prevent biofilm formation and disrupt mature biofilms at higher concentrations [34]. In addition, NAC has several beneficial properties, including mucolysis, scavenging hydroxyl radicals, modulation of inflammatory cascades, and collagenase inhibition, which supports its use as an adjunctive therapy in various ocular conditions [39, 40].

The objective of this study was to evaluate the minimal bactericidal concentration (MBC) and effective contact time of four topical antiseptics—PHMB, PVP‐I, HOCl, and NAC—against *Pantoea agglomerans* , cultivated from equine eyes with ulcerative keratitis.

## 2 Material and Methods

### 2.1 Sample Collection

Bacterial samples from corneal ulcers in horses were collected between August 2022 and January 2024. All swab samples from corneal ulcers of horses sent to the Institute for Microbiology, University of Veterinary Medicine Hannover, Foundation, Hannover, Germany, and to Laboklin GmbH & Co. KG, Bad Kissingen, Germany, were used to establish a pathogen collection. After 1 year of collection, less important bacteria were not further considered.

The inclusion criteria stated that the submitted swab had to be taken from an infected corneal ulcer of an equine eye, which in case of doubt was confirmed with the veterinarian who submitted the sample. From August 2022 to January 2024, the most frequently isolated species ( *Pantoea agglomerans* ) was selected for further processing and analysis.

Isolates were determined by culture and differentiation with matrix‐assisted laser desorption/ionization—time‐of‐flight mass spectrometry (MALDI‐TOF). Sensitivity patterns were tested for all selected isolates by microdilution using the breakpoint method established by the Clinical and Laboratory Standards Institute (CLSI). Information regarding any pre‐treatment and date of sampling were documented when available.

### 2.2 Minimal Bactericidal Concentration (MBC)

All available isolates from *Pantoea agglomerans* were processed by a single examiner in the same laboratory. The MBC of PHMB, PVP‐I, NAC, and HOCL were determined for all clinical samples collected during the specified sampling period.

MBC testing was conducted in accordance with CLSI *Methods for Determining Bactericidal Activity of Antimicrobial Agents (M26‐A)* [41] with minor modifications according to a recently established protocol [42]. Briefly, each isolate was tested against the four bactericidal substances in serial dilutions, with each dilution tested in triplicate. The following dilutions were used:

- PHMB: 6.4, 3.2, 1.6, 0.8, 0.4, 0.2, 0.1, 0.05 ppm
- PVP‐I: 64, 32, 16, 8, 4, 2, 1, 0.5 ppm
- NAC: 6400, 3200, 1600, 800, 400, 200, 100, 50 ppm
- HOCL: 6.4, 3.2, 1.6, 0.8, 0.4, 0.2, 0.1, 0.05 ppm

The stock solutions were prepared each morning by diluting a defined amount of each substance with sterile phosphate‐buffered saline (PBS): PHMB (polihexanid 20% solution, Fagron), PVP‐I (PVP1 Poly(vinylpyrrolidone)‐iodine complex, Sigma‐Aldrich), HOCL (Vetericyn.VF eye & ear solution, 0.275 g/kg; Innovacyn), and NAC (N‐Acetyl‐L‐cysteine, A7250, Sigma Aldrich). Then, serial dilutions were prepared in 2‐mL microtubes under sterile conditions.

Bacterial isolates were stored in glycerol at −70°C until further processing. Before testing, stored isolates were recultivated on Columbia blood agar (Columbia Blood Agar with Sheep Blood Medium, Thermo Scientific). For each of the three replicates, a suspension of each isolate was prepared in sterile PBS and adjusted to 0.5 McFarland standard using a densitometer. This suspension was serial diluted to yield a final bacterial concentration of 1.5 × 10^6^ cells/mL. Then 0.25 mL of bacterial suspension was added to 0.25 mL of each prepared dilutions of the four substances. After 10 min of incubation, 0.1 mL of inoculated suspension was transferred to 0.9 mL Dey‐Engley neutralizer (NutriSelect Plus) suspension, mixed with a vortexer at medium speed for 2 s, and allowed to neutralize for 5 min [42]. Subsequently, 0.1 mL of the neutralized suspension was applied onto Columbia blood agar, and colonies were manually counted after 16–24 h of incubation at 37°C.

A positive control (following the same procedure without bactericidal substance) was used to determine the maximum bacterial inoculum, and a negative control (following the same procedure without bacterial inoculation) was included to check for contamination.

MBC_10_ was defined as the lowest dilution at which 99.9% of inoculated bacteria were killed in all three replicates. Given an initial inoculum of 7500 CFU on each blood agar plate, < 8 visible colonies were consistent in achieving the MBC.

After determining the MBC, the effective contact time (kill‐time) was tested at one selected dilution above the MBC. The protocol described above was adjusted accordingly: the incubation period of bacteria with the bactericidal agent was set at 15 s, 30 s, 1 min, 2 min, 3 min, 4 min, and 5 min at one selected concentration. After incubation, a subset of the suspension was transferred to the neutralizer as described above. Each isolate was tested once against all substances. The kill‐time was defined as the time at which 99.9% of inoculated bacteria were killed. The evaluation of results was performed as previously described.

Since the NAC stock solution had an acidic pH, five isolates were tested in acidic conditions without a bactericidal substance to ensure that pH did not significantly affect the bactericidal effect. A PBS solution was adjusted to a pH of 2.2, comparable to the pH of the NAC stock solution. The MBC_10_ was assessed as outlined above.

The data analysis is purely descriptive. Microsoft Excel was used for this purpose. The figures were created with GraphPad Prism 9, GraphPad Software Inc.

## 3 Results

### 3.1 Bacterial Isolates

Over a 17‐month sampling period, a total of 141 bacterial isolates from 64 species were identified from 108 eyes of 107 horses. *Pantoea agglomerans* was the most frequently detected species (*n* = 16; 11%). Therefore, *Pantoea agglomerans* was chosen for all further examinations of this study. Of the 16 isolates detected, two failed to be collected due to a laboratory error, leaving 14 available for further analyses.

The isolates were obtained from horses of various breeds, including warmbloods (*n* = 5), ponies (*n* = 2), Andalusian horse (*n* = 1), Fjord horse (*n* = 1), Standardbred (*n* = 1), and horses of unreported breed (*n* = 4). The age of the horses ranged from 1 to 27 years. In two cases, *Pantoea agglomerans* was isolated in monoculture. In four cases, it was detected alongside other Gram‐negative isolates, including *Actinobacter bohemicus, Actinobacter virabilis*, and *Neisseria ssp*. In 10 cases, concurrent Gram‐positive isolates were also identified, including *Bacillus subtilis, Bacillus cereus, Paenarthrobacter ssp., Pseudoarthrobacter oxydans, Staphylococcus delphini, Staphylococcus vitulinus, Staphylococcus equorum, Staphylococcus gallinarum, Staphylococcus fleurettii, Staphylococcus sciuri, Streptococcus dysgalactiae*, and *Streptococcus mitis oralis*. Fungal culture was negative in seven cases and not performed in the remaining cases.

**FIGURE 1:**
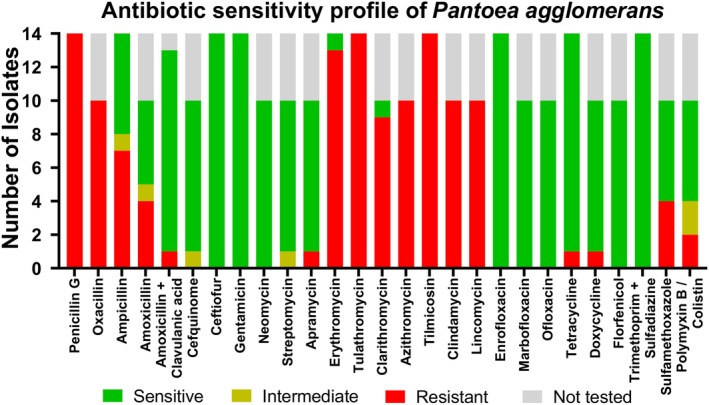
Overview of the resistance pattern of 14 *Pantoea agglomerans* isolates. ‘Not tested’ isolates resulted from different test substances in the two laboratories.

The tested *Pantoea agglomerans* isolates exhibited significant resistance to beta‐lactam antimicrobials, macrolides, lincosamides, and diaminopyrimidine. In contrast, cephalosporins, aminoglycosides, tetracyclines, fluoroquinolones, and diaminopyrimidine + sulfonamide combinations were effective against these isolates (Figure 1).

### 3.2 Minimal Bactericidal Concentration and Kill‐Time

#### 3.2.1 PHMB

**FIGURE 2:**
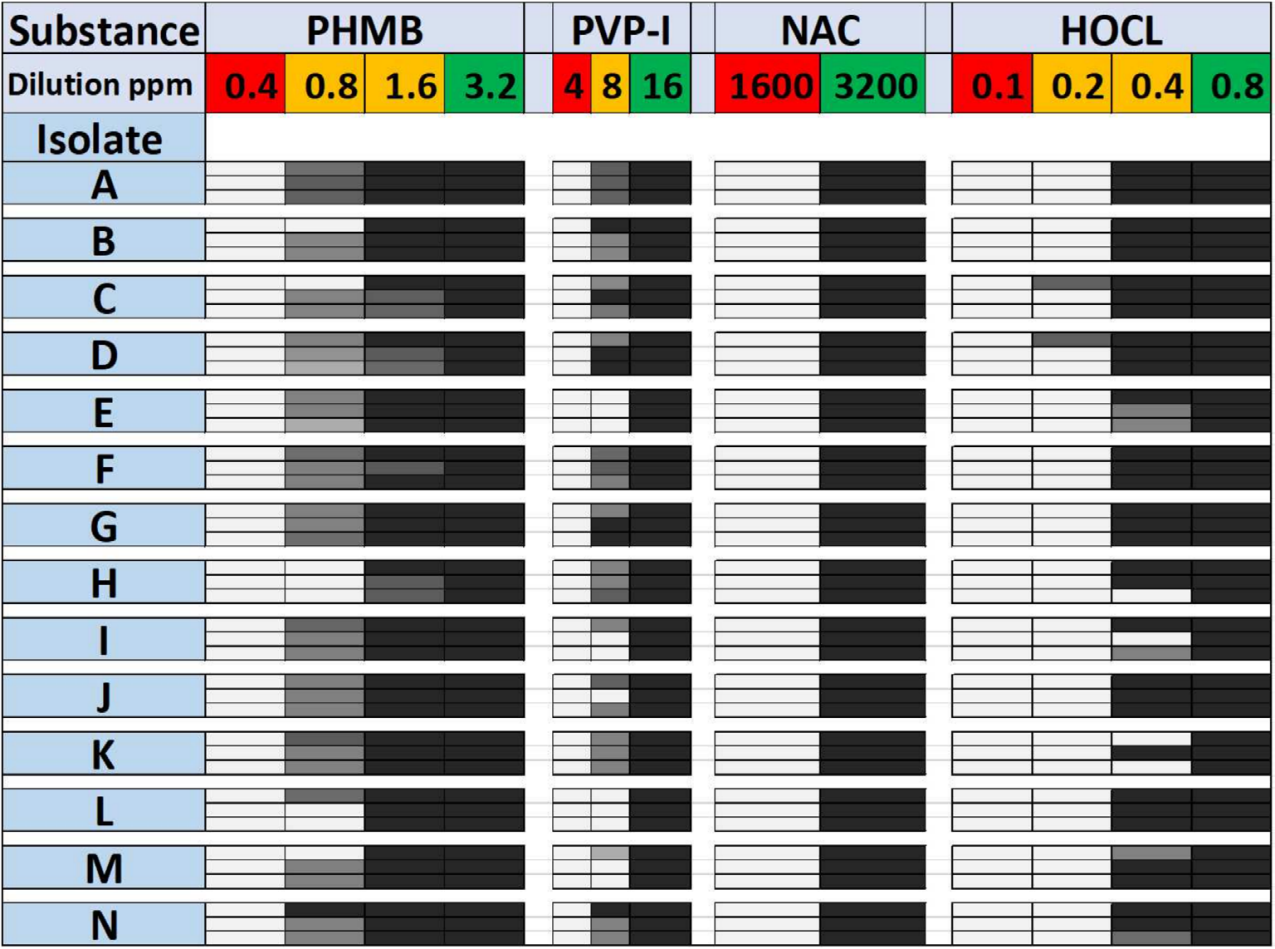
Graphical representation of the results of MBC testing for 14 isolates of *Pantoea agglomerans* (A‐N). Each isolate was tested in triplicate with the substances PHMB, PVP‐I, NAC, and HOCL. Three replicates are indicated by three rows per isolate. White fields indicate no reduction in CFU. Gray fields indicate a reduction in CFU that does not meet the MBC definition. Black fields fulfill the MBC definition. Only the relevant section of the serial dilution near the MBC is shown and given in ppm. The red color of the dilution indicates that no bactericidal effect was present for any isolate in that particular sample. The green color indicates that the MBC was reached for all isolates. Yellow concentrations had alternating results.

**FIGURE 3:**
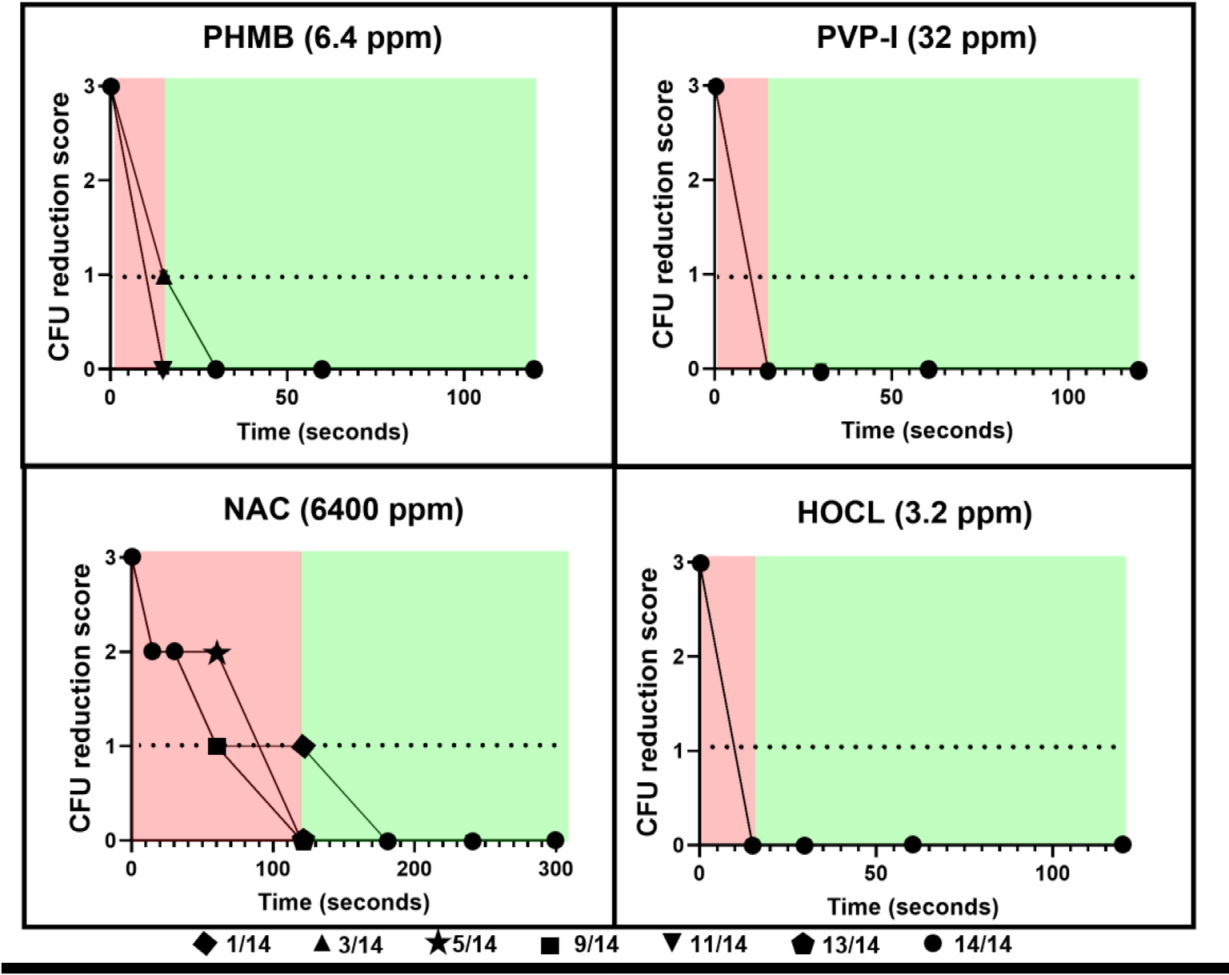
Graphical representation of semi‐quantitative analysis of the kill‐time of 14 *Pantoea agglomerans isolates* with four antiseptic agents: PHMB, PVP‐I, NAC, and HOCL. The x‐axis represents the time in seconds, while the y‐axis indicates the reduction in CFU. The y‐axis scale is defined as follows: 3 = no reduction in CFU, 2 = reduction in CFU without meeting the MBC criteria, 1 = reduction in CFU meeting the MBC criteria, 0 = no CFU detected. Different symbols indicate the fraction of pathogens at the same level at a given time. The symbol legend indicates the count of isolates displayed.

The MBC_10_ of PHMB for all *Pantoea agglomerans* isolates was 3.2 ppm (0.00032%), as this was the concentration at which the MBC_10_ was achieved in all replicates (Figure 2). One dilution lower (1.6 ppm), the MBC was achieved for 10 of 14 isolates. At 0.8 ppm, there was a reduction in CFU, but this did not meet the criteria for MBC. At 6.4 ppm, the time required to achieve the MBC (kill‐time) was 15 s (Figure 3). In three isolates, there was one colony detectable after an incubation period of 15 s.

#### 3.2.2 PVP‐I

The MBC_10_ for PVP‐I for all isolates was 16 ppm (0.0016%). At 8 ppm, the MBC was achieved for 2 of 14 isolates (Figure 2), and a reduction in CFU was detectable in most isolates (except two). At 32 ppm, the kill‐time for all isolates was 15 s (Figure 3).

#### 3.2.3 NAC

NAC reached its MBC_10_ at 3200 ppm (0.32%), with no reduction in CFU at the 1600 ppm dilution (Figure 2). The time‐kill at 6400 ppm was 2 min for all isolates (Figure 3). After 60 s, the MBC definition was not met in 5 of 14 isolates. At 30 s, the reduction in CFU did not meet the MBC definition in any isolate, although a decrease in CFU was consistently observed.

#### 3.2.4 HOCL

HOCL achieved a ≥ 99.9% reduction in CFU at concentrations of 0.8 ppm and above (Figure 2). At 0.4 ppm, the MBC_10_ was reached for 8 of 14 isolates, and a reduction in CFU was detectable in the remaining isolates. The kill‐time at 3.2 ppm was 15 s in all isolates (Figure 3).

After incubation of *Pantoea agglomerans* for 10 min in acidic circumstances, no reduction in CFU was detectable. All negative controls were negative, and positive controls showed consistent growth of bacterial colonies.

## 4 Discussion

Given the increasing antimicrobial resistance to commonly used first‐line antibiotics in equine ophthalmology [1], there is a critical need for effective alternatives. Moreover, a reduction in the use of antibiotics is also urgently required in line with the One Health approach [43]. A comprehensive study has shown, for example, that antibiotic use in food‐producing animals can increase antimicrobial resistance that represents a threat to humans; conversely, a reduction in antibiotic use to the minimum level is required in order not to threaten the health of animals and humans in the long term [43]. It is also known that local antibiotic therapy can influence the normal ocular microbial flora [7, 44] or favor pathogenic organisms [45]. Even if this effect has not been shown consistently in horses [7, 46], it should be taken into account when using antibiotics on the ocular surface.

Our study focused on clinical isolates of *Pantoea agglomerans* , identified as the most frequently occurring species in equine ulcerative keratitis in our equine population during the sampling period. This finding may highlight the emerging significance of this pathogen in equine ocular conditions, as suggested by recent studies. In Belgium, for instance, *Pantoea agglomerans* was reported as the most commonly isolated gram‐negative pathogen in cases of equine ulcerative keratitis [1]. Similarly, a comprehensive laboratory survey investigating the ocular microflora of equine eyes with various, unspecified diseases in Germany found 256 isolates of *Pantoea agglomerans* from 844 samples, which may further support its relevance [11]. Previous studies on microbial pathogens in ulcerative keratitis from other regions have rarely [3, 4] or never [5, 47] detected *Pantoea agglomerans* . Interestingly, *Pantoea agglomerans* has also been recently detected in the eyes of healthy donkeys in Poland [15]. It is therefore unclear at this point whether this pathogen has a regional prevalence or whether it has become more common in recent years.

In human medicine, *Pantoea agglomerans* is recognized as a facultative pathogen [48, 49] and has been implicated in contact lens‐related corneal ulceration [14]. Its common presence in plant material [48, 49] may explain how horses come in contact with this bacterium. The spread of this pathogen is also possible via dust from plant material, as has been shown by studies with grain dust and other agricultural dust, where *Pantoea agglomerans* was frequently detected and is also associated with various diseases (e.g., lung diseases) in human medicine [49]. Given the frequent isolation of this strain in this study and the limited focus of previous studies on it, this study specifically targeted *Pantoea agglomerans* .

Limited data are available regarding the antimicrobial susceptibility of *Pantoea agglomerans* isolated from equine eyes to antibiotics commonly used in the treatment of equine ulcerative keratitis. Previous studies that frequently isolated *Pantoea agglomerans* from equine eyes [1, 11] reported combined sensitivity patterns for all Gram‐negative isolates or on a *family* basis. Their findings indicate significant resistance to beta‐lactam antibiotics, with sporadic resistance to fluoroquinolones, tetracyclines, and polymyxins. Consistently, our study demonstrated that *Pantoea agglomerans* exhibited marked resistance against beta‐lactam antibiotics, which aligns with its characteristics as a Gram‐negative bacterium. However, all tested isolates were susceptible to fluoroquinolones, which is particularly relevant given their common use in topical therapy of severely infected ulcers. Tetracycline, a frequently employed first‐line antibiotic, was effective against most isolates, with resistance observed in only one case. In human medicine, it is evident that *Pantoea agglomerans* is a pathogen that has the potential to develop multiple drug resistance [50]; even if usually only sporadic resistance occurs. Nonetheless, resistance to beta‐lactam antibiotics in particular is high [51].

Therefore, the choice of antimicrobial treatment should be guided by susceptibility patterns, and the use of antibiotics should be minimized whenever possible. It should be noted that the sensitivity tests are based on the effective levels in distinct tissues [52]. Due to a lack of data, it is not possible to draw reliable conclusions for efficacy of local ocular therapy. Given that no currently available antibiotic can be effective against all typical eye pathogens, and the choice of treatment in the face of an infected corneal ulcer must be made before culture and susceptibility results are available, it is essential to investigate potential alternatives to their use. For this purpose, we initially used *Pantoea agglomerans* , the pathogen most frequently detected in our study, to compare the efficacy of four different antiseptics.

This study demonstrated the effectiveness of all four tested bactericidal substances.

PHMB, PVP‐I, and HOCL were effective at lower concentrations compared to NAC against *Pantoea agglomerans* .

The MBC_10_ of PHMB in this study aligns with reported minimal inhibitory concentrations in other isolates from humans, ranging from 0.1 to 25 ppm [53], and is lower than those in the presence of fetal blood serum [54]. PHMB showed a time‐ and concentration‐dependent mode of action [55, 56]. The kill‐time in this study was faster than observed in previous studies on, for example, *S. aureus* [56]. However, data on its effectiveness specifically against *Pantoea agglomerans* have not yet been reported.

Given that PHMB is well tolerated, effective in the presence of proteins [53], and available in formulation with sufficient concentrations to achieve therapeutic levels in ocular application, it shows promise as an alternative to reduce the need for antibiotics in treating ulcerative keratitis. Notably, PHMB has been shown to become more effective than PVP‐I when prolonged contact time is possible, while PVP‐I reaches its maximum effect fast [56]. In our study, both substances had a bactericidal effect on the pathogen examined after 15 s so that no differentiation was possible with the method used here.

PVP‐I was highly effective against *Pantoea agglomerans* . It is known to be more effective in diluted than in concentrated solution [57]. At appropriate concentrations, the bactericidal effect was achieved within 15 s [57]. The MBC_10_ in this study was 16 ppm, which is higher than the MBC_24_ of *S. aureus* but lower than the range reported for other bacteria [56]. The rapid effectiveness of PVP‐I demonstrated in this study is particularly relevant for local ocular therapy. Prolonged contact time did not enhance the bactericidal properties, as noted in another study. Given the potential cytotoxicity to the corneal epithelium [21], a short contact time and appropriately low concentration are essential. The MBC_10_ determined here was more than 1000‐fold lower than the reported toxic concentration in other species [21].

HOCL also proved effective against *Pantoea agglomerans* at low concentrations. As a naturally occurring component of the innate immune system [23], its tissue compatibility is potentially superior to synthetic agents, supported by its high therapeutic index against Gram‐negative bacteria [23]. Consistent with previous reported time‐kill rates of less than 1 min [23], the short contact time required was confirmed in our study. The good tissue compatibility and short required contact time make this substance an appropriate choice for local ocular treatment.

While NAC is not primarily recognized as an antiseptic agent, recent studies have highlighted its potential in this context [34, 36]. In this study, NAC demonstrated a bactericidal effect, but compared to the other substances examined it was effective at higher concentrations and longer contact times. The MBC_10_ observed here is within the range of MIC reported in previous studies on isolates from small animals, with MIC_24_ values between 1560 and 6250 ppm [36]. Despite the limited understanding of its effects at the microstructural level, this study contributes to the growing evidence that NAC possesses antimicrobial properties. Given its various beneficial effects in local ocular therapy and low toxicity [39], NAC is beneficial in treating equine ulcerative keratitis. Although its bactericidal effect may not be the primary rationale for its use, maintaining a therapeutic concentration for the required contact time should be achievable, considering tear volume (233.74 μL) and turnover rates (13.21%/min) in equine ocular fluids [58].

As pure substances rather than commercially available ocular formulations of PHMB, PVP‐I, and NAC were used to prevent the influence of adjuvants, varying pH values were observed in stock solutions. Since NAC was the only solution with an acidic pH, its effect was assessed separately. Testing with five randomly chosen isolates, we confirmed that pH alone did not have a bactericidal effect, which is important, as commercial NAC products have added adjuvants that may alter the pH value.

Overall, PHMB, PVP‐I, and HOCL demonstrated potent bactericidal effects at low concentrations against *Pantoea agglomerans* cultured from equine ulcerative keratitis. Based on the MBC_10_, the four tested substances can be categorized according to their in vitro effectiveness against *Pantoea agglomerans* : HOCL > PHMB > PVP‐I > NAC. The time‐kill values clearly show which substances have the fastest effect: HOCL = PVP‐I = PHMB << NAC.

Examples of commercially available formulations of the investigated antiseptics are many times higher concentrated than the determined MBC_10_. In the case of NAC, the commercial Stromease 25 mg/mL (DOMES PHARMA) is slightly more than seven times more concentrated than the MBC_10_. This is even more pronounced for HOCL (Vetericyn.VF; 220 times more concentrated than MBC_10_) and for PHMB (Lavasept, B.Braun; 125 times more concentrated than MBC_10_). If PVP‐I is used in a 1% dilution, however, the concentration is 625 times higher than the MBC_10_. For each of the substances tested, there are products available that are licensed for use on the ocular surface.

The significantly higher concentration of antiseptics has the advantage that it could compensate for possible interfering factors. An interference with protein occurs, especially with PVP‐I [16] and HOCL [23]. Since proteins are present in the tear film of healthy and diseased equine eyes [59], the efficacy of these antiseptics must still be evaluated in vivo. Furthermore, it is recommended that an eye be cleaned prior to the application of antiseptic agents to minimize the potential for interaction with mucus or proteins.

Our results support clinical studies investigating PHMB, PVP‐I, and HOCL as suitable disinfectants for the ocular surface, not only in preparation for surgery, but also for short‐ and medium‐term treatment of infectious conditions. HOCL may be superior in vitro; however, which of the tested substances will prove more suitable in vivo depends on tissue tolerance and the influence of interfering factors such as proteins and should be investigated for the equine eye. To determine the clinical benefit of the tested antiseptics, their effect on the microflora of healthy eyes and on pathogens in corneal ulcers should be investigated.

The bactericidal effect of NAC may also be an additional positive effect in the treatment of infected corneal ulcers when used as an adjuvant.

The study has several limitations. Most clinical cases of corneal ulcerations were not examined by any of the authors, as samples of external laboratories were included. This allowed for a comprehensive collection of isolates but limited detailed information concerning the clinical presentation.

The frequent occurrence of *Pantoea agglomerans* in combination with other pathogens may suggest that it requires predisposing factors to cause disease, similar to what has been observed in human medicine [14, 48]. Nonetheless, considering other reports [1, 11, 12] and its high prevalence, we believe there is clinical relevance to this bacterium.

Additionally, the efficacy of antiseptics in infectious keratitis needs to be verified with other clinical isolates to better justify the empirical use of these substances. Our research group is currently investigating.

The methodology used in this study differed in some respects from standard guidelines for MBC testing [41], primarily because a feasible protocol was already established in the laboratory [42]. The transferred volume for CFU counting was slightly lower than the 10 CFU recommended for the MBC cutoff. The guidelines recommend cation‐adjusted Mueller Hinton Broth, but PBS was chosen to eliminate the risk of interaction between the reagents and the broth, as discussed above, with focus on interaction between proteins and antiseptics. This approach was proven feasible in a previous study [42]. Additionally, no reference strain was tested, as there was no available reference stain from the ocular surface, and we chose to focus solely on clinical isolates.

Another limitation to consider is the low correlation between in vitro MBC or time‐kill values and in vivo results, as noted in the CLSI guidelines [41]. Therefore, the promising results of this study need to be validated on the horse's cornea, either ex vivo or in vivo.

## 5 Conclusion

This study emphasizes the potential significance of *Pantoea agglomerans* in equine ulcerative keratitis. All tested antiseptic substances, PHMB, PVP‐I, NAC, and HOCL, demonstrated effectiveness against *Pantoea agglomerans* . PHMB, PVP‐I, and HOCL are promising alternatives to antibiotics in the treatment of equine infectious keratitis and may help to reduce the use of antibiotics or even improve the resistance situation. This study provides the basis for further evaluating their use on the surface of the equine eye.

## Author Contributions

**Frederik Heun:** conceptualization, writing – original draft, methodology, investigation, validation, formal analysis, visualization. **Jessica Meißner:** methodology, resources, writing – review and editing, supervision. **Ann‐Kathrin Schieder:** investigation, data curation, writing – review and editing. **Bernhard Ohnesorge:** resources, supervision, project administration, conceptualization, writing – review and editing. **Claudia Busse:** project administration, writing – review and editing, conceptualization, supervision.

## Ethics Statement

No ethical approval was required for this study, as all studies were performed using bacterial isolates obtained as part of the clinically directed assessment.

## Conflicts of Interest

The authors declare no conflicts of interest.

## Data Availability

The data that support the findings of this study are available from the corresponding author upon reasonable request.
